# What Evidence Exists for Initiatives to Reduce Risk and Incidence of Sexual Violence in Armed Conflict and Other Humanitarian Crises? A Systematic Review

**DOI:** 10.1371/journal.pone.0062600

**Published:** 2013-05-15

**Authors:** Jo Spangaro, Chinelo Adogu, Geetha Ranmuthugala, Gawaine Powell Davies, Léa Steinacker, Anthony Zwi

**Affiliations:** 1 School of Social Sciences, University of New South Wales, Sydney, Australia; 2 Australian Institute of Health Innovation, University of New South Wales, Sydney, Australia; 3 Centre for Primary Health Care and Equity, School of Public Health and Community Medicine, University of New South Wales, Sydney, Australia; 4 Search for Common Ground, Bukavu, Democratic Republic of Congo; Tulane University, United States of America

## Abstract

Sexual violence is highly prevalent in armed conflict and other humanitarian crises and attracting increasing policy and practice attention. This systematic review aimed to canvas the extent and impact of initiatives to reduce incidence, risk and harm from sexual violence in conflict, post-conflict and other humanitarian crises, in low and middle income countries. Twenty three bibliographic databases and 26 websites were searched, covering publications from 1990 to September 2011 using database-specific keywords for *sexual violence* and *conflict* or *humanitarian crisis*. The 40 included studies reported on seven strategy types: i) survivor care; ii) livelihood initiatives; iii) community mobilisation; iv) personnel initiatives; v) systems and security responses; vi) legal interventions and vii) multiple component interventions. Conducted in 26 countries, the majority of interventions were offered in African countries. Despite the extensive literature on sexual violence by combatants, most interventions addressed opportunistic forms of sexual violence committed in post-conflict settings. Only one study specifically addressed the disaster setting. Actual implementation of initiatives appeared to be limited as was the quality of outcome studies. No studies prospectively measured incidence of sexual violence, although three studies provided some evidence of reductions in association with firewood distribution to reduce women's exposure, as did one program to prevent sexual exploitation and abuse by peacekeeping forces. Apparent *increases* to risk resulted from lack of protection, stigma and retaliation associated with interventions. Multiple-component interventions and sensitive community engagement appeared to contribute to positive outcomes. Significant obstacles prevent women seeking help following sexual violence, pointing to the need to protect anonymity and preventive strategies. This review contributes a conceptual framework for understanding the forms, settings, and interventions for conflict and crisis-related sexual violence. It points to the need for thorough implementation of initiatives that build on local capacity, while avoiding increased risk and re-traumatisation to survivors of sexual violence.

## Introduction

Sexual violence in armed conflict, despite being documented through history [1], gained attention in the 1990 s following widespread assaults of women in Rwanda and the former Yugoslavia. These abuses prompted United Nations (UN) interventions [2] and led to five resolutions by the UN Security Council; these recognised sexual violence as a tactic of war, called for accountability, and established monitoring mechanisms [3], [4], [5], [6], [7]. Current evidence suggests 4%–22% of women experience sexual violence in conflict [8], although the data are considered incomplete [2], [9], [10]. Reports consistently point to children and women as the primary targets, but it is recognised that men are also, at times, victims [11], [12], [13]. Globally, sexual violence has profound impacts on physical and mental health, including injuries, HIV and other infections, sexual and reproductive health problems, deaths as a result of suicide, murder or ‘honour killings,’ maiming, stigmatization, and ostracism by families and communities [14]. Additional recorded effects in times of conflict include forced pregnancy, elevated rates of traumatic fistula [15], abandonment of children conceived through rape, cultural destruction and exacerbated stigma when the assailant is a hostile combatant [16].

Sexual violence is understood as a *sexual act committed against a person, or in which a person is caused to engage in sexual acts by force, threat of force or coercion such as that caused by fear of violence, duress, detention, psychological oppression or abuse of power, or by taking advantage of a coercive environment or a person's incapacity to give genuine consent* [17]. Attention has also been drawn to sexual violence in disaster and post-disaster settings, with a three-fold increase reported in Sri Lanka after the Indian Ocean tsunami [18], and similar spikes following the 2010 Haiti earthquake [19]. Armed conflict, post-conflict settings and disaster all share high rates of population displacement along with breakdown of the social and legal systems that deter criminal behaviour [10]. All three settings pose risks for sexual exploitation and abuse, the umbrella term for acts by peace keepers and humanitarian workers who abuse positions of power and trust for sexual purposes in emergencies [20]. Low and middle income countries (LMICs) are disproportionately affected by both conflict and other humanitarian crises, with fewer resources to respond, thereby warranting particular attention.

A proliferation of policy and other initiatives aimed at addressing sexual violence in crisis followed the UN Security Council resolutions [21], [22], [23], [24], [25], [26], [27]. A key development was the introduction of the International Criminal Court (ICC) in 1998, based on the legal code known as the Rome Statute, following judgements by the International Criminal Tribunals for the former Yugoslavia and Rwanda. The effectiveness of the ICC in prosecuting and deterring future crimes is uncertain [28]. Other legal measures to address conflict-related sexual violence include sign off by selected countries on aligning criminal codes with the Rome Statute and use of tribunals such as the Gacaca community courts, in Rwanda [29].

It is timely to consider the impact of these initiatives. Studies on the scope and effectiveness of programs to prevent and respond to conflict-related sexual violence have been identified as research priorities [30]. Although one review of the prevalence of sexual violence in conflict has been published [8], the evidence for interventions to reduce or prevent sexual violence have not been systematically reviewed to date. The current review, commissioned by the Australian Agency for International Development (AusAID) of the evidence for reducing risk or incidence of conflict and crisis-related sexual violence begins to address this gap.

### Aim of review

This review sought to answer the over-arching question: *What is the evidence of the impact of initiatives to reduce risk and incidence of sexual violence in conflict and post-conflict states and humanitarian crises in low and middle-income countries?*

Sub-questions were:

1. What evidence exists for implementation of interventions to reduce sexual violence?
2. What evidence exists for reduced *incidence* of sexual violence as a result of interventions?
3. What evidence exists for reduced *risk* of sexual violence as a result of interventions?
4. What evidence exists for the secondary prevention interventions to reduce the impact of sexual violence on survivors?

These questions recognize the low likelihood of identifying reduced incidence as a consequence of an intervention, due to both the difficulty in establishing causation in a complex area and recognition of the challenges of conducting research in crisis settings. Accordingly, the questions are deliberately broadly based and include as outcomes, evidence of implementation of interventions as well as for reduced risk of sexual violence.

### Conceptual framework for understanding sexual violence and interventions

Sexual violence in conflict and crisis is a complex social and legal problem with multiple contributors for which a range of interventions from diverse disciplines have been developed and deployed. Through preliminary review of the literature we developed a conceptual framework to guide the review in relation to the forms and settings in which sexual violence in conflict, post-conflict and disaster occurs, as well as the type of interventions. We identified four forms or contexts in which sexual violence occurs. *Militarized sexual violence* is a deliberate and systematic means of terrorising and humiliating communities [10] and may be instituted as a form of “ethnic cleansing” [31]. It is not always possible to determine whether this is the intent behind sexual violence by combatants and not all sexual violence in conflict takes this form. *Opportunistic sexual violence* involves a broader range of perpetrators who take advantage of opportunities presented by individuals being unprotected by family or statutory agencies and an environment of impunity to commit acts of sexual violence. Perpetrators include armed militias, national or invading military forces, international peace-keeping troops, security or border personnel, humanitarian workers or bandits. *Sexual abuse and exploitation* (SEA) by peacekeepers or humanitarian staff, may be considered a third form of sexual violence in these settings: where personnel abuse their positions of power and trust for sexual purposes, for example through exchanging emergency relief supplies for sexual acts. Finally, in every community, sexual violence pre-dates emergencies but is *exacerbated* by heightened gender inequalities [32], breakdown of regular norms, additional stresses on relationships [24], and attempts by men to reassert control [33].

Drawing on the ecological model for understanding contributors to intimate partner violence [34], a second dimension of our conceptual framework identifies nine strategy types which can be understood as operating at the individual, community or societal level, as described in Table 1. Individual level strategies are directed at individuals, community level strategies are operated by or engage whole communities, and societal level strategies in general work at a higher level, implemented by state bodies or international agencies.

**Table 1:** Strategies for addressing sexual violence in conflict and crisis.

	Strategies and examples
**INDIVIDUAL**	**Survivor responses**: Provision of medical and/or psycho-social care, forensic assessment of survivors and advocacy
	**Livelihood strategies:** Provision of training and/or support (eg.micro-finance) to women to increase their economic independence and reduce their vulnerability to sexual violence and/or provide rehabilitation post sexual violence
	**Combatant-focused initiatives:** Disarmament, demobilisation and reintegration (DDR) programs that target reduction of sexual violence or rehabilitation of survivors assaulted during capture by combatants; engagement with combat leaders
**COMMUNITY**	**Community mobilisation:** Promotion of reporting; education of rights in regard to sexual coercion; increasing opportunities for women to participate in political, economic and social activities; human rights education; engagement with men and boys on human rights, including gender equality
	**Peace building for prevention of sexual violence:** Incorporation of sexual violence prevention measures in ceasefire negotiations and monitoring; also inclusion of women in negotiating bodies
**SOCIETAL**	**Personnel:** Use of codes of conduct, training on attitudes/protocols/responses with military/peacekeepers/police/aid workers; policies to reduce opportunity by personnel for sexual exploitation and abuse; deployment or increased recruitment of female officers
	**Systems and security:** Provision of foot and vehicle patrols/security details to vulnerable areas; establishment of safety protocols,eg firewood patrols or distribution to reduce vulnerability
	**Infrastructure:** Segregation of water/sanitation facilities; construction of shelters/schools
	**Legal action**: Specialist prosecution units/tribunals; initiatives involving community or customary justice systems; and indictments through the International Criminal Court.

Each of these strategies may be implemented separately or as part of multiple component interventions. This review includes survivor care strategies aimed at addressing effects of sexual violence on individuals. While not having a direct preventive effect this type of intervention is often part of forensic examination used for prosecution, which is itself a potentially important strategy for deterrence [35]. Furthermore, provision of responses to victims, may operate as secondary prevention, that is, reduced *harm* as a result of the event.

Our conceptual framework also included measures for reduced risk, which entailed a set of indicators listed below which we identified as an analytical tool, prior to the searching.

### Review indicators for reduced risk

- Reduced incidence/Increased sense safety in community
- Care to survivors results in improved wellbeing (secondary prevention)
- Reintegration/livelihood programs to survivors reduces exposure to sexual violence (SV)
- Combat leaders engaged to halt SV
- Disarmament, Demobilisation and Reintegration (DDR) programs implemented targeting SV
- DDR programs include safety/livelihood programs for women/girls
- Women in peace-building targeting SV
- Awareness of rights by community
- Awareness of availability of services/reporting mechanisms
- Willingness/uptake of services/reporting mechanisms
- Increased awareness by men in community of equal rights and impact of abuse
- Implementation/impact of codes of conduct/training
- Gender specific (ie. female) recruitment implemented
- Disciplinary action initiated
- Coordination mechanisms established
- Impact of patrols/firewood alternatives
- Completion of situational analysis of risk of sexual violence
- Impact of infrastructure designed for risk reduction
- Systems for distribution of food/other resources established for reduction of SEA
- Legal action initiated/convictions
- Country action on International Criminal Court provisions

These indicators reflect the type of interventions being deployed in the field and built on recommendations for monitoring and evaluating gender-based violence (GBV) initiatives [36].

In light of this being a relatively new field of practice, the need to understand the diversity of practice and the low likelihood of quality research being conducted in crisis settings, we adopted an inclusive approach to this review, incorporating empirical and grey literatures identified from scientific and academic databases, web sites and targeted searches.

## Method

This project was informed by realist approaches to research and systematic reviews [37]; this aspect of the analysis will be covered in detail separately. The review was registered with EPPI-Centre, a British registration body for reviews of social and health interventions with a focus on interventions in low to middle income countries through which the review protocol is available [38]. We established and were advised by an international reference group to source insights from potential review users.

### Search Strategy

We examined peer-reviewed journal articles and published and unpublished grey literature with a publication date between January 1990 and August 2011. A comprehensive search strategy of 23 bibliographic databases was undertaken : Medline (PubMed on Ovid), CINAHL, PsycInfo, PAIS, Global Health, ASSIA, Gender studies, Violence & Abuse Abstracts, Wageneingen University Disaster Studies, Proquest Dissertations & Abstracts, Lexis-Nexis, UNICEF Children in armed conflict, GDNet Knowledge Base, African Journals Online, 3ie database of impact evaluations, Bibliomap & TRoPHI (EPPI-Centre), World Health Organization Library (WHOLIS), EBM Reviews, Cochrane Database of Systematic Reviews, Johanna Briggs systematic review and Campbell Collaboration databases. Search terms for bibliographic databases were “sexual violence” and “conflict” or “post-conflict” or “humanitarian crisis” and their synonyms. As an example the Medline search strategy was. *“abused women[tw] OR “abused woman”[tw] OR “forced sex”[tw] OR “enforced sex”[tw] OR gbv[tw] OR “gender based violence”[tw] OR rape[tw] OR raped[tw] OR rapist[tw] OR raping[tw] OR “sexual abuse”[tw] OR “sexual coercion”[tw] OR “sexual violence”[tw] OR “sexual assault ”[tw] OR “sexual exploitation”[tw] OR “sexual slavery”[tw] OR “violence against women”[tw] OR “unwanted sex”[tw] OR “unlawful sex”[tw] OR “sexual exploitation and abuse”[tw] “militarised sexual violence”[tw] OR “forced pregnancy”[tw] OR “enforced pregnancy” [tw] AND “armed conflict”[tw] OR “armed incursion”[tw] OR “post conflict”[tw] OR “human security”[tw] OR “war zone”[tw] OR coup[tw] OR invasion[tw] OR insurrection[tw] OR “peace keeping”[tw] OR “peace building”[tw] OR “child soldiers”[tw] OR “boy soldiers”[tw] OR “internally displaced persons”[tw] OR “displaced populations”[tw] OR “displaced persons”[tw] OR “refugee camps”[tw] OR “humanitarian response”[tw] OR “humanitarian assistance”[tw] OR “humanitarian crisis”[tw] OR “humanitarian crises”[tw] OR “post-crisis” [tw] OR “post-crises”[tw] OR war[mh] OR Refugees [mh] OR Disasters [Mesh:noexp] OR Disaster Planning [mh] OR Mass Casualty Incidents[mh] OR Relief Work[mh] OR Rescue Work[mh] OR Avalanches[mh] OR Earthquakes[mh] OR Landslides[mh] OR Tidal Waves[mh] OR Tsunamis[mh] OR Volcanic Eruptions[mh] OR Fires[mh] OR Cyclonic Storms[mh] OR Floods[mh].*

Twenty three websites searched were: *HRH Global Resource Center* http://www.hrhresourcecenter.org/, *Sexual Violence Research Initiative web site* http://www.svri.org/, *UNWomen* http://www.unwomen.org/, *End violence against women* http://www.endviolenceagainstwomen.org.uk/, *United Nations Population Fund)* http://www.unfpa.org/public/, *Gender and Disaster Network* http://www.gdnonline.org/, *United Nations High Commission on Refugees* http://www.unhcr.org/cgi-bin/texis/vtx/home, *Stoperapenow* http://www.stoprapenow.org/advocacy-resources/index/?t=10&p=1. *Women's Initiatives for Gender Justice* http://www.iccwomen.org/, *GBV One response* http://gbv.oneresponse.info, *Gender-based Violence Network: Essential Tools for GBV Prevention and Response in Emergencies* http://www.gbvnetwork.org/, *Bridge (* http://www.bridge.ids.ac.uk/ *), JOLIS (World bank and IMF library catalogue)* http://jolis.worldbankimflib.org/e-nljolis.htm, *USAID* http://www.usaid.gov/, *Overseas Development Institute* http://www.odi.org.uk/resources/, *ELDIS* http://www.eldis.org/, *Governance and Social Development Resource Centre* http://www.gsdrc.org/, *International Centre for Research on Women* http://www.icrw.org/icrw-library, *International Development Research Centre* http://publicwebsite.idrc.ca/EN/Pages/default/aspx, *Public Policy Pointers* http://www.policypointers.org/, *British Library Development Studies catalogue* http://blds.ids.ac.uk/search-the-collection, *Women's International League for Peace and Freedom International Rescue* http://womenpeacesecurity.org/members/wilpf/, *International Committee of the Red Cross* http://www.icrc.org/eng/, *UN Disarmament, Demobilisation and Reintegration Resource Centre* www.unddr.org/index.php, *Reproductive Health Response in Crisis (RHRC) Consortium's GBV in conflict online bibliography* http://www.rhrc.org/resources/gbv/bib/index.cfm?category=prev.

In addition three journals were hand searched: *Violence Against Women; Medicine, Conflict and Survival* and *Disasters* which were identified by team and Advisory Group members as likely sources of eligible studies. In addition we consulted key informants including the nine members of our Advisory Group in respect of journals, data bases and web sites to search. We also searched the reference lists of identified studies.

### Screening of studies

Inclusion and exclusion criteria as presented in Table 2, were agreed by the team and then applied by authors JS and CA to titles and abstracts. Full reports were obtained for those studies that met the criteria based on title and abstract screening, or where insufficient information was provided. The inclusion and exclusion criteria were then re-applied to the full reports and those that did not meet the criteria were excluded. Titles were excluded where there was no abstract and the title and other provided information indicated that the paper was out of scope. A sample of 50 titles was screened by two team members; agreement was 96% (Kappa analysis 0.65) indicating good agreement between those searching and classifying the papers [39]. Uncertain instances were discussed jointly by both screening team members and if not resolved by the other co-authors (AZ, GR, GPD) as a group.

**Table 2:** Inclusion criteria for screening of studies.

	INCLUDED	EXCLUDED
TOPIC	Sexual violence in the context of conflict or humanitarian crisis.	Studies that did not address sexual violence of women, men or children. Studies that addressed female genital mutilation, trafficking, enforced sterilisation, and/or HIV prevention.
TYPES OF STUDIES/DATA	Studies containing primary empirical data describing the implementation or impact of interventions including: cross-sectional surveys, prospective or retrospective single group or comparison group designs, evaluations including formative evaluation, case studies, qualitative studies based on interview or focus group, policy analysis, field data.	Studies describing only the nature and extent of the problem; or barriers to implementation of, or access to, interventions generally; or interventions that were not specific to sexual violence; papers that mentioned interventions without any descriptive information or primary empirical data that described the implementation or impact of interventions.
TYPES OF PARTICIPANTS	Survivors of sexual violence, combatants, peacekeepers, humanitarian workers, community members, camp residents, service providers.	Commentators or actors not directly involved in implementation of interventions
TYPES OF INTERVENTIONS	Interventions which aimed at reducing the incidence of or risk of sexual violence, including secondary and tertiary prevention of sexual violence.	Interventions that did not make reference to reduction of sexual violence as a specific aim or outcome (e.g. DDR programs/peace-building programs where this aim was not explicit), or interventions aimed at HIV prevention.
SETTINGS	Context of conflict, post-conflict or other humanitarian emergency in lower and middle-income countries.	Context of *the intervention* was not conflict/post-conflict or humanitarian crisis as identified by the author(s) in title/abstract. Interventions not conducted in the specific context of conflict/post-conflict or humanitarian crisis (eg. school interventions). Countries not included on the World Bank List of low/middle-income countries in 2010.
TYPES OF PUBLICATIONS	Research papers or research/descriptive reports published since 1990.	Letters, editorials, comment, periodicals, review, editorials, art works, news updates, speeches that were not published in journals.
LANGUAGE OF PUBLICATION	English. Where study titles and abstracts were in English, but manuscripts were in a foreign language and met other inclusion criteria, translation was considered.	Study titles and abstracts in a language other than English.
PUBLICATION DATE	January 1990-August 2011	Studies published before 1990.

### Analysis of results

Using a standardised template, data were extracted on: country of intervention, strategy type, target population, study design and participants, organisation type, context, reported outcomes and unintended consequences. A further dimension was whether studies reported outcomes or described only implementation of interventions. Implementation studies included those that provided primary empirical data as listed in Table 2. Outcome studies included those which described the impact of interventions, including perceptions of service users, where these were relevant to risk or incidence. Studies were rated for decrease or no change to risk or incidence of sexual violence. Narrative analysis considered each strategy type against the review sub-questions, that is: evidence for i) implementation of interventions to reduce sexual violence; ii) reduced *incidence* of sexual violence; iii) reduced *risk* of sexual violence as a result of interventions; and iv) impacts of secondary prevention interventions on survivors wellbeing. Unanticipated negative effects of interventions and apparently relevant contextual factors were also noted. Data were managed using the EPPI-Centre software *EPPI-Reviewer*.

#### Quality assessment

Included studies were individually assessed for quality [40], [41] by JS and CA jointly. Accordingly ratings of high, medium or low in respect of:

A: Soundness of method (extent to which a study is carried out according to good practice within the terms of that method);

B: Appropriateness of study type to answer the review question (appropriateness of methods to the review question);

C: Relevance of the topic focus for the review question;

D: Overall weight of evidence that can be attributed to the results of the study.

The overall ratings are determined as *High* (A, B and C all rated as high); *Medium* (A, B and C all rated as either medium or high, with subcategories of medium-high if one or two rated as high, or medium-low if one rated as low) and *Low* (two or more rated as low) [40], [41]. Soundness of method for qualitative studies was guided by the COREQ guidelines for assessing reporting of qualitative research [42].

Recognising that richness of data also provides valuable insights, studies were also ranked according to the qualitative measure of “thick” or “thin” description alluding to the depth and availability of contextual information provided [43], [44], [45]. Studies were classified as comprising “thick” description if lucid information on program components was provided, such as staffing, as well as factors affecting program implementation or outcomes. “Thin” descriptions were those in which these components were lacking or weak. This measure was undertaken to ensure that each study was fully considered for its value to the review in light of the inclusion of implementation data. Studies were not excluded on the basis of quality or depth of description, but these were taken into account in conclusions drawn. Feedback from our Reference Group, EPPI-Centre, and peer reviewers were also incorporated.

## Results

### Studies Identified

**Figure 1:**
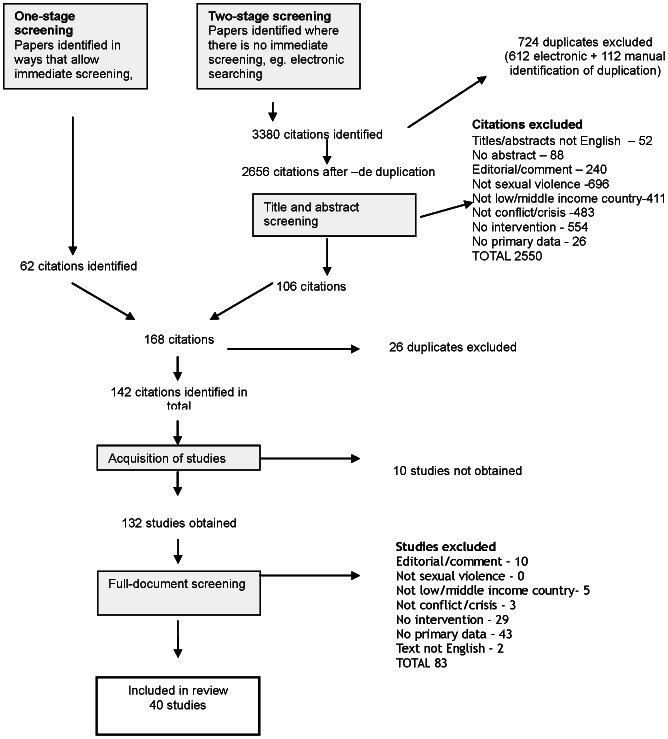
Filtering of studies.

The search strategy yielded 2656 citations (after removing duplicates) from 23 bibliographic databases and 26 websites. In addition, studies were identified from: hand search of three journals (1), review of citations lists (0) and key informants (5). Of the 40 included studies, 21 were identified from websites, one from a key informant and the remaining 18 through bibliographic databases. Figure 1 reports the filtering process.

#### Study type

Twenty studies reported outcomes and the other 20 studies reported only on the implementation of these different types of interventions.

#### Country of study

Interventions were undertaken in 26 countries, predominantly in Africa and the former Yugoslavia with Liberia, Rwanda and Kenya being the sites with most studies (four each). Three of these studies reported interventions in multiple countries. Apart from these, two studies were undertaken on global implementation of initiatives (defined here as more than 5 countries).

#### Setting

The majority of studies (24) addressed interventions for sexual violence occurring in post-conflict settings. Fourteen studies addressed conflict settings, one study addressed interventions for both conflict and post-conflict settings [46] and another addressed interventions for sexual violence in all three setting types [47].Only one study addressed the disaster context specifically [48].

#### Nature of sexual violence addressed

Considering our conceptual framework eighteen studies reported on interventions targeting more than one form of crisis related sexual violence. Of those that addressed a single form, 14 addressed militarised sexual violence, three addressed SEA, another three addressed exacerbated community sexual violence. Two studies specifically addressed opportunistic sexual violence.

#### Agencies delivering interventions

Half of the studies described interventions delivered as partnerships between different types of organizations. The biggest providers of interventions were international non-government organisations (21/74 agencies), followed by multi-lateral agencies (18/74), local NGOs (15/74) and state agencies (11/74), community groups (4/74), international tribunals (3/74) and state tribunals/courts (2/74).

#### Strategy type and target population

Seven of the nine different types of strategy initially identified and defined in Table 1 were the subject of the included studies. The largest group were those combining multiple strategy types (13), followed by survivor care strategies (10), legal interventions (6), community mobilization, personnel, systems and security strategies (3 each) and livelihood programs (2). Multiple strategy interventions included two or more different strategy types as part of an integrated sexual violence response. No studies addressed initiatives for women in peace building or combatants, despite these being identified as potential preventive strategies in the literature. Also largely missing were infrastructure initiatives, apart from as minor elements in two studies. Reflecting the strategy types, the largest target group receiving interventions was survivors of sexual violence, followed by communities, either as a whole or in specific groups, predominantly women, but also men and children or combinations of these.

Table S1 provides summary data on all 40 studies including country of study, type of crisis, form of conflict/crisis related sexual violence, intervention, target population, study method and study group. Studies in italics and marked (O), report on outcomes, the remainder report only on implementation of interventions.

### Evidence of implementation of initiatives

Inclusion of studies that reported only on implementation, in addition to outcome studies, provided the opportunity to understand the scope of interventions currently being employed. The following section provides an overview of each strategy type from both of these categories of included studies.

#### Survivor care strategies

Survivor care responses predominantly comprised either medical, advocacy and counselling assistance for survivors of rape, [42], [47], [49], [50], [51] which may include follow-up psychotherapy [52] or shelter [53]. A second prevailing model is provision of support groups [54], [55]. Use of traditional rituals delivered by community elders, aimed at exonerating individuals and creating a break with the past, appeared to have important potential. The girls and boys to whom it was delivered were both survivors and perpetrators of crimes while part of the Lord's Resistance Army in Uganda [56]. High levels of psychological distress among the young people were documented and the rituals were seen to be important in removing their shame and culpability. The authors unfortunately did not attempt to measure the impact of the rituals. The use of a confidential hotline [57] and extending service provision to those who had experienced other types of war trauma such as torture and loss [52], were measures aimed at increasing the anonymity and therefore accessibility of services to survivors.

#### Livelihood strategies

Livelihood strategies are assumed to prevent violence against women through increasing decision-making in the home and financial independence [58]. Of the two studies, one based on case histories of girls using a disarmament, demobilisation and reintegration (DDR) program for former child soldiers, found poor protection of girls in the camps [59], though minimal data was provided on the the program. The second study evaluated a program for refugee women in Cairo involving vocational training, job placement and monitoring of security of women in their work place [58]. The program was criticised by the study authors for failing to directly address gender-based violence.

#### Community mobilisation strategies

Three studies mobilised communities to prevent sexual violence through: awareness groups provided to men in Burmese refugee camps [60]; engaging community groups to make and screen short films about gender-based violence [61]; and strengthening local leadership to respond to and prevent sexual and gender-based violence [62]. The studies lacked detailed information on how the community was engaged and none reported outcomes, however they demonstrated involvement with men, including local leaders via targeted activities.

#### Personnel strategies

Three studies reported personnel strategies, two of which targeted sexual exploitation and abuse for peacekeeping forces [63] and humanitarian workers [64]. A third focussed on gender-specific police reform in Kosovo, Liberia and Sierra Leone [65]. Gender-specific police reform is supported by the UN as a means to enhance community security aiming to reduce sexual and other forms of GBV. The logic appears to be that female officers will both support women's confidence in making reports, as well as potentially reducing threats from male officers, though this was not made explicit in the report. Strategies included setting a quota for female recruitment (Sierra Leone set at 30%), training and appointment into leadership roles (Liberia, Kosovo) and establishment of a safe environment for women, along with child support policies (Kosovo) [65].

#### Systems and security strategies

Three papers described firewood distribution, patrols and provision of alternative fuels, aimed at preventing opportunistic sexual violence associated with women leaving refugee camps to gather firewood [66], [67], [68]. In general the target population for firewood and/or fuel interventions are women, who typically are those responsible for fuel collection. In some instances strategies may include men, for example encouraging them to accompany women or to take on the task of firewood collection.

#### Multiple strategy interventions

The most common components in these strategies were survivor responses (12/13) and community mobilization (9/13) studies. Personnel initiatives and systems and security initiatives were also commonly combined with these first two strategy types. The services provided are similar in profile to those described in the preceding sections. Most interventions included one or more activities employing community mobilisation, most commonly: awareness raising workshops among women and sometimes male community members, as well as engagement of community leaders. Other activities included training of community volunteers, and the introduction of a community-wide alcohol ban and local night patrols. Personnel strategies included training of local health workers and police and, introduction of codes of conduct (CoC).

Findings from the multi-component interventions point to the value of community participation as a means to promote service uptake. An example was the Guinean program which recruited community members for a two tiered response to Sexual and GBV [69]. Initially ‘community trainers’ were trained and deployed in refugee camps to raise awareness to the point where the residents would request a ‘community worker’ position. These workers received more extensive training and took on more complex tasks of establishing local committees, constructing safe spaces and providing counselling [69]. The same initiative also addressed community tensions about imposing a ‘gender equality’ lens, by having equal representation of men and women in the planning stages. An alternate approach to preventing resentment by male community members towards gender-rights focussed work, was taken by a program in Sierra Leone for women and girls abducted by combatants. The program extended their services to children and partners of the women [70], providing them with free medical care, education and childcare. One program that aimed to address problems with centralized provision of expert services shifted to mobile outreach providing social work, gynaecological and psychiatric care, simultaneously training local health workers [71]. Recognizing the risks of undermining local initiatives by introducing imported Western models of care, staff incorporated traditional healers to address spiritual dimensions of trauma.

#### Legal strategies

Seven studies reported on provision of legal initiatives including one paper which also reported on a livelihood initiative [59]. These initiatives cluster around Rwanda and countries within the former Yugoslavia, which have both been sites of specialist legal tribunals for sexual violence in conflict.

These findings on the seven strategy types provide an overview of programs being implemented on the ground. Overall, despite the extensive work undertaken in developing policy, guidelines, training programs and legal responses, five studies concluded that actual implementation of interventions remained flawed or limited [49], [72] [67], [64], [73]. Three of these were those with the highest weight of evidence as reported in Table 3.

**Table 3:** Weight of evidence and depth of description among outcome studies.

Author(s)	Weight of Evidence	Depth of Description
Gruber 2005	Medium-Low	Thick
Hustache et al 2009	Medium	Thin
Manneschmidt & Griese 2009	Low	Thick
Zraly & Nyirazinyoye 2010	Medium-Low	Thin
Denov 2006	Medium-Low	Thin
Jennings 2008	Medium-Low	Thick
Lattu 2008	Medium-High	Thick
CASA Consulting 2001	Medium-High	Thick
Women's Commission for Refugee Women 2006	Medium-Low	Thin
Bizarri 2010	Medium	Thick
Blogg, Hickling et al 2004	Medium-High	Thin
Schei & Dahl 1999	Low	Thin
Women's Commission Refugee for Women 2009(b)	Low	Thin
UNHCR 1998	Low	Thick
UNHCR 1997	Low	Thick
Brouneus 2008	Medium-Low	Thick
Human Rights Watch Africa 1996	Medium-Low	Thin
Mischkowski & Mlinarevic 2009	Medium	Thick
Nowrojee 2005	Medium-Low	Thick
Denov 2006	Medium-Low	Thin
Women's Initiative for Gender Justice 2010	Medium-Low	Thick

### Quality of studies

As reported in Table 3, quality of evidence assessment undertaken for the 20 studies that reported outcomes found that 14 had a low or medium-low Weight of Evidence (WOE) rating. No studies had a WOE rating greater than medium-high. In considering the level of description provided 12 studies were rated as constituting “thick” description, and eight “thin” description. Two of the three studies which were of medium-high WOE also provided thick description [64], [67], other study results showed more variation across these two tools as indicated in Table 3. In respect of evidence for incidence, risk and secondary harm, the quality of studies and risks for bias point to the need for caution, given that on all measures, strength of evidence must be considered low.

### Evidence for reduced incidence of sexual violence

Table 4 reports on the results of those studies which described outcomes of the implementation in terms of whether risk and incidence were reduced (↓), increased (↑), not reported (NR) or no change (NC). None of the studies set out to systematically and prospectively address the impact on incidence of sexual violence as a result of interventions. As reported in Table 4 three studies did provide some evidence of this in the form of reduced victim reports of sexual violence in association with firewood distribution/fuel alternatives [67] (WOE: Medium-High), [66] (WOE: Medium) and a program to prevent SEA by peacekeepers [63] (WOE: Medium-Low). In respect of the SEA program, the apparent decline in incidence was reported only in one of the two sites studied. Victim reports are an unreliable measure, as an increase can indicate improved confidence by victims in responses, rather than an actual increase in incidence, however in each of these studies the apparent decline in incidence was supported by key informant's views. There was no evidence for reduced incidence as a result of other interventions, although some, such as legal interventions, are long term measures in this respect.

**Table 4:** Outcome studies: Changes, to risk, incidence and other effects from interventions to address sexual violence.

Author(s)	Change to Risk	Change to Incidence	Other effects
Gruber 2005	Not Reported (NR)	NR	Lack of uptake of service which was opposed by partners and other men in community
Hustache et al 2009	NR	NR	Improved functioning in 39% of 178 survivors 1–2 yrs post intervention; Significant decrease in impairment (p = 0.04)
Manneschmidt & Griese 2009	NR	NR	Of 137 participants from 12 groups immediately post–intervention 36% reported reduced distress
Zraly & Nyirazinyoye 2010	NR	NR	Post-conflict women's support groups for SV or other issues can create support networks.
Denov 2006	↑ (Increased)	NR	Increased vulnerability for SV for girls in DDR camps due to poor protection, over-crowding and lack of enforcement of rules
Jennings 2008 Liberia & Haiti	↓ (Decreased) Liberia NR Haiti	↓ Liberia NR Haiti	LIBERIA: Increased reporting following high profile cases in 2005,followed by apparent decreased incidence HAITI: No promotion of confidential hotline to community. Alternative report mechanism not confidential
Lattu 2008	No Change (NC)	NC	All 3 countries, very low reporting, no community consultation or information provision on SEA intervention, low awareness of reporting mechanisms
CASA Consulting 2001 Kenya	↓ (firewood collection)	↓ (firewood collection)	45% decrease in rapes during firewood collection, possibly due to employment opportunities for local and refugee men -Simultaneous increase of 78–113% in other contexts and locations
Women's Commission for Refugee Women 2006	↓	NR	After training, 1/3 women were capable of making fuel-efficient mud stove ; reduced frequency of firewood collection (from 4× to 1× weekly)
Bizarri 2010	↓	↓	NGOs report decline as a result of fuel provision, awareness-raising in schools, improved reporting and support mechanisms
Blogg, Hickling et al 2004 Uganda Congo	↓ Uganda ↓ Congo	NR Uganda NR Congo	*UGANDA -* Increased awareness of GBV through sensitisation-perceived decrease in cases reported by informants *CONGO- Betou -* Extensive GBV community leader training program (270 attended 4 sessions) Drop-in centre - 20% cases for rape- decline of cases noted since height of crisis *CONGO -Brazzaville -* Evidence of strong uptake of clinic
Schei & Dahl 1999	NR	NR	Handcrafts group - regular users of the Centre had lower PTSD symptoms than non-users (53% vs 68%: n = 209); Therapy group –Group completers lower PTSD than non-completors (69% vs 81%: n = 158) (incomplete data and no statistical analysis)
Women's Commission Refugee for Women 2009(b)	↓ firewood collection only	NR	Provision of stoves to 90% households reduced need to collect firewood Livelihood programs did not deter violence due to: increased exposure to partner abuse to hand over earnings, risks posed by firewood gathering for sale, exposure to risks as domestic workers in towns
UNHCR 1998	↓ Kibondo NC Kasulu	NR	*Kibondo -* High uptake survivor service (1000 users in 12 mo);-facilitated by “drop in” model *Kasulu -* Low uptake (23 users during 15 mo)– Men opposed strategies to reduce women's risk
UNHCR 1997 Tanzania	↓	NR	Increased reporting of rapes (from 4 to 7 per month) ;-introduction of community identified risk reduction strategies
Brouneus 2008	↑	NR	Interviews with 16 women who testified in Gacaca courts: Testifying re-traumatising & all experienced attacks, threats or destruction of property after wards; Lack of protection
Human Rights Watch Africa 1996	NR	NR	ICTR −80,000 cases awaiting trial at date of publishing; Under-reporting due to lack of confidence, lack of female personnel, lack of protection, fear of stigma and retaliation
Mischkowski & Mlinarevic 2009	NR	NR	Interviews with 49 survivor-witnesses ICTY or War Crimes Chamber-Bosnia & Herzogovina;-Testifying traumatic ; Dismissive treatment by investigators and prosecutors (both) ;Lack of confidentiality during and after trials (both)
Nowrojee 2005	↑	NR	7/21 cases completed by ICTR included rape charges 1/7 resulted in conviction; Lack of confidentiality & follow-up protection; proceedings exacerbated trauma
Denov 2006	NR	NR	Sierra Leone Truth & Reconciliation Commission–witnesses unwilling to testify; Special Court Sierra Leone - Prospect of appearing induced fear of reprisal
Women's Initiative for Gender Justice 2010	↓	NR	ICC - Slow progress against gender parity in recruitment; Women comprise 58% of judges, 18% of legal counsel, 23% of field staff and 47% overall ;-SV charges included in 6/10 cases before the court; Only 27% of SV survivor applicants granted leave to participate since outset

### Evidence for reducing risk of sexual violence

On the basis of our indicators and as reported in Table 4, reduced risk was not reported for survivor care programs. The single livelihood program which reported outcomes appeared to *increase* risk, through lack of protection provided for the girls in the DDR camp [59] (WOE: Medium-Low). In respect of personnel strategies efforts to address SEA do not on the whole appear to be associated with reduced risk for this form of abuse. This may result from poor community engagement, lack of safe and confidential means of reporting assaults coupled with inadequate follow-up, described by studies [63] (WOE: Medium-Low) and [64] (WOE: Medium-High). One exception was the site where reduced incidence was also documented, following action on cases which then received widespread publicity, apparently prompting increased reporting by victims [63].

There is some evidence that firewood provision, patrols and alternative fuels have reduced risk during firewood collection but not necessarily in other situations at the same sites [67]. Study authors pointed to the need for integrated multiple component interventions to address this likelihood [67] (WOE: Medium-High), [66](WOE: Medium), [68] (WOE: Medium-Low). Although the weight of evidence for the studies that employed multiple component interventions was mostly low, there was support for this approach, with four of the five outcome studies in this category demonstrating reduced risk in the form of evidence of willingness to use services, assured access to fuel and/or increased community awareness of rights [73] (WOE: Medium-High), [74] (WOE: Low), [75] (WOE: Low), [76] (WOE: Low).

Indicators for risk were mixed in relation to legal interventions. One study presented findings that on the one hand sexual violence charges were included in the majority of cases currently before the ICC (6/10), but only 27% of survivors had been granted leave to participate [77] (WOE: Medium-Low). Other studies found low rates of prosecution of sexual violence in both the International Criminal Tribunal for Rwanda and the Gacaca courts [78] [79] (WOE: Medium-Low).In another study, participants reported that they believed their risk was reduced through giving evidence which flagged that sexual violence was wrong, and held perpetrators accountable [80] (WOE: Medium). On the other hand there were indicators that in some instances risk has *increased*, through retaliation, lack of protection and ostracism [29], [79], (WOE: Medium-Low). Further evidence was reported that that survivors find testifying traumatic [59] (WOE: Medium-Low) [80], (WOE: Medium) and preparation by prosecution staff was lacking [78], [79], [80] (WOE: Medium-Low).

### Evidence for secondary prevention (reduced harm)

In terms of secondary prevention, there is some evidence that providing a medical response and two sessions of post-trauma counselling improves functioning that is sustained 1–2 years later [51] (WOE Medium) and that opportunities to participate in groups and build networks with other women who have experienced trauma are valued by women and may reduce distress even where sexual violence is not the explicit focus [54], [55].

### Contextual factors influencing outcomes

Community engagement in the design and delivery of interventions appeared to contribute to the success of programs. Eight of the ten studies reporting reduced risk or incidence targeted the community as a whole, or discrete groups within the community; four of these employed community mobilisation strategies. Active community engagement was evident at the sites in four of the ten studies in which risk or incidence was reduced [73] WOE: Medium-High) [74], (WOE: Low) [75], (WOE: Low) [76], (WOE: Low). These studies also employed multiple strategies however, and it is not possible to determine the relative impact of the various aspects of the intervention.

In respect of survivor care programs, anonymity of access appears to contribute to higher service uptake by survivors [73] (WOE: Medium-High) [51], (WOE: Medium),) [54], [76], (WOE: Low). However significant cultural and economic obstacles prevent many women from reporting or seeking help following experiences of sexual violence. This occurs where women risk divorce or loss of marriage prospects, if identified as a victim of sexual violence. This may explain why strategies that do not rely on reports by victims to be activated, such as patrols or firewood alternatives, seem to have greatest success. In relation to other potential contextual factors no patterns were identified with respect to the influence on outcomes of: conflict vs. crisis setting; country; whether or not countries were signatories to the Rome Statute, or sex of the survivor. The lack of trend may reflect insufficient evidence rather than a lack of relevance of these factors.

## Discussion

This review identified seven different strategies being implemented in 26 countries to address incidence, risk and secondary prevention of conflict and crisis related sexual violence. Based on available studies it appeared that the greatest activity is in African countries, post-conflict settings, targeting opportunistic forms of sexual violence with interventions directed to survivors of sexual violence. The evidence points to overall limited implementation of initiatives and the quality of outcome studies was low. No studies prospectively measured impacts on incidence of sexual violence, although three studies provided some evidence of reductions in association with firewood distribution to reduce women's exposure as did one program to prevent sexual exploitation and abuse by peacekeeping forces. These interventions predominantly target opportunistic sexual violence, as opposed to militarized or community-based violence. Apparent *increases* to risk resulted from lack of protection, stigma and retaliation, particularly in respect of legal interventions. Sensitive community engagement appeared to contribute to positive outcomes although significant cultural and economic obstacles prevent many women seeking help following sexual violence.

Limitations of the study included that no bibliographic database for literature on military interventions was identified which may have meant that studies in this discipline were missed. Similarly studies about interventions that did not explicitly refer to addressing sexual violence, or to conflict, post-conflict, crisis or refugee settings in the key words, title or abstract may not have been identified. The search may also have missed studies with titles and abstracts in languages other than English. Single screening by two team members may have resulted in varied application of the criteria, although co-location allowed for constant discussion and comparison and inter-rater reliability was 96%. The cross-sectoral nature of interventions conducted with divergent aims, practices and standards, restricted synthesis, as did the disparate target groups and study designs, lacking comparative data for the most part. Inclusion of grey literature resulted in inclusion of self- reporting by agencies with high risk of bias. Limited data were available on changes to risk, and our adopted indicators may have over-rated reductions in risk.

Many of these limitations are to be expected, given the fraught and unstable nature of crisis zones in which conducting sound research will continue to remain highly challenging. As has been observed, conducting a valid and reliable study of sexual violence is difficult in any setting, but compounded in humanitarian settings where fear, stigma, secrecy and displacement create additional barriers [8]. In that light, this project makes a number of important contributions to the field.

Firstly, it contributes a conceptual framework for understanding the forms of conflict and crisis-related sexual violence and interventions being implemented. Secondly the review maps actual implementation of the many launched initiatives by multi-lateral agencies, states and large and small non-government organizations across 26 countries, highlighting the clustering and gaps in contexts, settings and target groups. While none show clear evidence for reduced incidence and evidence for reduced risk is weak, this was to be expected and key emergent themes, gaps and contextual factors have been identified.

Ours appears to have been the first systematic review to address interventions across this field however our findings align with those by other authors on the limited nature of evidence for reduced incidence [2], [9], [30]. Similarly the limitations we found in terms of the quality of evidence have also been noted previously [8]. The barriers to reporting sexual violence and the lack of support experienced by survivors who choose to testify in courts align with other findings [81]. A key contextual factor we identified as important to the success of interventions, which was the need for community engagement, has also been noted by others, particularly in the form of building trust with civil society organizations and engaging workers and residents in shared problem solving [82] [83].

In considering the adequacy of evidence found in this review, it must be remembered that the evidence for prevention of sexual violence is still in its early days in *all* settings, quite apart from in conflict and other crises. A global review by the World Health Organization found no interventions with proven effectiveness for sexual violence, noting that *many strategies appear to have potential, either on theoretical grounds or because they target known risk factors, but most of these have never been systematically implemented – let alone evaluated* [84] (p.6).

Despite the fact that the literature and UN action in this area focuses on sexual violence during conflict, most often, militarized sexual violence, our findings point to a clustering of interventions in post-conflict settings, predominantly addressing opportunistic sexual violence. This form of violence also appears to be more amenable to intervention, than the three other forms of sexual violence we identified. This may because combatants, peace keepers and intimates have greater coercive power. While the firewood patrols are often held up as successful examples of interventions for conflict, these target opportunistic violence, lending support to this idea. Additionally, we found that the majority of interventions target survivors, rather than other structures or institutions. Not surprisingly legal interventions were not associated with reduced risk or incidence, in the short term however their important role in ending impunity is sufficient argument for inclusion in the mix of interventions. As well as highlighting the gaps for target populations, settings and forms of sexual violence, we identify continuing barriers and some negative consequences for survivor-witnesses that are cause for concern.

### Implications for policy, practice and research

This review reinforces the importance of multifaceted interventions, highlighting the importance of strengthening such multi-layered interventions and evaluating them carefully.

Funded programs should incorporate robust outcome evaluation at the highest standard possible prioritising inclusion of community member perspectives, including those of survivors. Furthermore the findings support the need for interventions that strengthen local capacity, seeking to build on pre-existing systems and traditions within communities where possible. Systems for holding offenders accountable that require survivor participation, through reporting/giving evidence, need to be designed to privilege survivors needs for protection and avoidance of re-traumatisation. The lack of identified interventions addressing combatants is insufficient reason to discount the value of prevention directed to these groups. This could build on research on the Mai Mai militia in DRC finding diverse motives by combatants for committing sexual violence, with possibilities for intervention tailored to these motives and the different decision making structures of armed groups [85]. Similarly, opportunities should be explored for peace building strategies specific to preventing sexual violence such as inclusion of women in negotiating cease-fire arrangements and making sexual violence a topic of negotiations, for which no studies were identified. Above all the review points to the need for thorough implementation of interventions.

In considering the research agenda in this field while recognising the constraints of crisis settings, priority should be given to well designed, independent studies on the impact and contextual factors that influence interventions that employ robust comparative measures and provide clear definitions, in particular on settings for interventions, extent and nature of interventions, nature of violence and sex and age groups of populations. Further research is needed on how traditional healing and associated culturally sensitive strategies can be best harnessed to reduce and address sexual violence.

## Conclusion

This first systematic review of the evidence for reduced risk, incidence and secondary prevention of sexual violence in the context of conflict and other crises, provides a broad-based and multi-sectoral overview of the state of implementation and outcomes from efforts in this field. Contributing to the debates through analysis of documented experience to date, this review also engages with the story behind these stories. This has highlighted, not surprisingly, the importance of appreciating context, and of ensuring that if effective outcomes are to be achieved, multiple activities and strategies should be offered as part of interventions, because they are mutually reinforcing and enabling, and together offer some hope that prevention of sexual violence in conflict, post-conflict and other humanitarian crises is possible.

## Supporting Information

Table S1 Overview of all included studies.
(DOCX)
