# Supplementary material for: What Evidence Exists for Initiatives to Reduce Risk and Incidence of Sexual Violence in Armed Conflict and Other Humanitarian Crises? A Systematic Review
Source: PLoS One. 2013 May 15;8(5):e62600. doi: 10.1371/journal.pone.0062600 (PMC3655168; doi:10.1371/journal.pone.0062600)
Supplement: Table S1 — Overview of all included studies. (DOCX) [file pone.0062600.s001.docx]

## *Table S 1 Supplementary Table : Overview of Studies*

| **Study** | **Country** | **Crisis**  **Type** | **Nature of SV** | **Activities** | **Target population** | **Methodology** | **Study Group** |
| --- | --- | --- | --- | --- | --- | --- | --- |
| **SURVIVOR CARE STRATEGIES** | | | | | | | |
| *O Gruber (2005)* | *Eritrea* | *Conflict* | *Militarised SV* | *Medical and counselling assistance to SV survivors* | *Survivors of SV* | *Qualitative interviews* | *Women survivors of SV Health workers* |
| *0 Hustache et al (2009)* | *Congo* | *Conflict* | *Militarised SV* | *Post-rape psychological support (median 2 sessions) following medical assessment/treatment* | *Survivors of SV* | *Before & after; Cohort* | *Women survivors of SV* |
| *0 Manneschmidt & Griese (2009)* | *Afghanistan* | *Post conflict* | *Militarised SV*  *Exacerbated SV* | *Psycho-social group counselling for Afghan women affected by war and domestic violence* | *Survivors of SV* | *Qualitative focus group* | *Women survivors of SV* |
| *0 Zraly & Nyirazinyoye (2010)* | *Rwanda* | *Post Conflict* | *Militarised SV* | *Mutual support through advocacy/self help groups* | *Survivors of SV* | *Qualitative interviews* | *Women survivors of SV* |
| Amone-P'Olak (2006) | Uganda | Conflict | Militarised SV | Traditional rituals to cleanse victims /perpetrators atrocities | Survivor/ combatants | Implementation description/data | N/A |
| Chynoweth (2008) | Jordan | Post- conflict | Militarised & Opportunistic SV, SEA, Exacerbated SV | Extent of implementation of Minimum Initial Service Package (MISP) for reproductive health (RH) | Survivors of SV | Field visits | N/A |
| Common & Deodens (2004) | Global | ConflictPost-conflictOther crisis | Militarised SV | -Provision of MISP Distribution and use of RH kits | Survivors of SV | Cross-sectional survey of field offices - users of RH kits | Personnel |
| Horn (2010) | Kenya | Post- conflict | Exacerbated SV | UNHCR and community responses to intimate partner violence (IPV) | Community Members: women | Qualitative focus group | Community members: Women and men |
| Skjelsbæk (2006) | Bosnia and Herzegovina | Conflict | Militarised SV | Individual psychotherapy with war rape survivors | Survivors of SV | Qualitative interviews | Personnel |
| Mrsevic (1997) | Serbia | Post- conflict | Exacerbated SV | Survivor assistance and counselling provided via phone | Survivors of SV Women and children | Implementation description data | Survivors of SV- women |
| **LIVELIHOOD STRATEGIES** | | | | | | | |
| *O Denov (2006)*  **also reported in Legal* | *Sierra Leone* | *Conflict* | *Militarised SV* | *Disarmament, demobilisation and reintegration (DDR)* | *Survivor/ combatants* | *Field visits* | *Young women service users* |
| Women’s Commission for Refugee Women and Children (2009a) | Egypt | Post- Conflict | Opportunistic SV SEA | Livelihood program for refugee women | Community members: Women & Survivors of SV | Field visits | Women Community members |
| **COMMUNITY MOBILISATION STRATEGIES** | | | | | | | |
| Alvarado & Paul (2007) | Burma | Post- Conflict | Exacerbated SV | Groups engaging men in attitude change around GBV | Community members:Men | Implementation description/ data | N/A |
| Molony et al (2007) | Liberia | Conflict | Militarised & Opportunistic SV SEA Exacerbated SV | Community training and production of local video to promote prevention and reporting to services | Whole community | Implementation description/ data | N/A |
| UNHCR (2001b) | Liberia | Post- conflict | SEA Exacerbated SV | Capacity building of service providers law enforcement, judiciary and refugee-camp based community leaders to respond to and prevent SV Community engagement: women and men | Whole community Local personnel | Implementation description/data | N/A |
| **PERSONNEL STRATEGIES** | | | | | | | |
| *O Jennings (2008)* | *Haiti Liberia* | *Post- conflict* | *SEA* | *Zero-tolerance policy for SEA Training Code of Conduct (CoC), curfews, staff to wear uniforms at all times, fraternalising discouraged*  *Hotline for reporting* | *Whole community Survivors of SV Personnel* | *Field visits* | *Community members: Men and women; Survivors of SV Personnel* |
| *O Lattu (2008)* | *Kenya Namibia Thailand* | *Post conflict* | *SEA* | *Zero-tolerance policy for SEA Strengthening and staff training on CoC Complaint boxes Community education Community participation in producing film about SEA (Kenya only)* | *Whole community Personnel* | *Qualitative interviews* | *Community members: Men, women and Children* |
| UNDP UNIFEM (2007) | Kosovol Liberia  Sierra Leone | Post-conflict | Opportunistic SV SEA | Police recruitment Quotas of female officers Deployment in pairs/ groups across units Family-work policies | Personnel | Implementation description/ data | N/A |
| **SYSTEMS & SECURITY STRATEGIES** | | | | | | | |
| *O CASA Consulting (2001)* | *Kenya* | *Post conflict* | *Opportunistic SV* | *Distribution of firewood in Dadaab refugee camp* | *Community members: Women* | *Cross-sectional survey Field visits* | *Survivors of SV- Women; Personnel* |
| *O Women’s Commission for Refugee Women and Children (2006)* | *Sudan* | *Post- conflict* | *Opportunistic SV SEA* | *Provision of fuel efficient stoves, firewood & alternative fuels using patrols* | *Community. members: Women, men and children^[[1]](#footnote-1)^* | *Implementation description/dataQualitative interviews & focus group* | *Community members: Women; Personnel* |
| *O Bizarri (2010)* | *Kenya* | *Post Conflict* | *Opportunistic SV* | *Provision of firewood & fuel-efficient stoves; Establishment of reporting mechanism* | *Community. members: Women* | *Multiple data case studies* | *Community members: Men and women Personnel* |
| **MULTIPLE COMPONENT INTERVENTIONS** | | | | | | | |
| *O Blogg et al (2004) Strategies: Survivor (S)+ Community Mobilisation (CM)* | *Uganda Congo* | *Post conflict* | *SEA Exacerbated SV* | *Medical /counselling support/ legal information for survivors Engagement of community leaders Community alcohol ban, curfew & night patrols* | *Community. members: Women* | *Field visits* | *Community members: Men and women* |
| Rees et al (2005) *Strategies: S+CM* | Sri Lanka | Disaster | Opportunistic SV Exacerbated SV | Support groups to address trauma and engage women as community advocates | Community members: Women | Implementation description/ data | N/A |
| UNHCR (2001a) *Strategies*: S+CM | Guinea | Post-conflict | Opportunistic SV SEA Exacerbated SV | Coordination and advocacy Community awareness raising, provision of shelter & counselling- establishment of local advocacy committees -equal representation of men and women in planning | Whole community Survivors of SV Personnel | Implementation description/data | N/A |
| Bracken et al (1992) *Strategies: S + Personnel (P)* | Uganda | Conflict | Militarised SV Opportunistic SV | Survivor counselling & medical care Health worker training Support groups Recognition of traditional healers | Survivors of SV Personnel | Implementation description/data | N/A |
| *O Schei & Dahl (1999) Strategies: S + P* | *Bosnia and Herzegovina* | *Conflict* | *Militarised SV* | *Counsellor training Recreational/craft group compared to weekly psychotherapy group (3-4 months)* | *Survivors of SV* | *Comparison two models Before and after surveys* | *Community members: Women* |
| *O UNFPA (2006) Strategies: S + Livelihood Strategies (Li) + Legal Action (Le)* | *Sierra Leone* | *Post-conflict* | *Militarised SV SEA* | *Service for women/girls abducted by combatants Sexual and reproductive health, counselling, legal advice, shelter Sensitisation and health services also for partners and children ,Vocational training* | *Community members: Women, men and children Survivors of SV* | *Implementation description/data* | *N/A* |
| Doedens et al (2004) *Strategies: System & Security Initiatives (Sys) + P* | Chad | Post-conflict | Opportunistic SV SEA | Staff training on CoC Some attention to latrine/ water design and food distribution to reduce SV | Personnel | Field visits | Whole community Personnel |
| *O Women’s Commission Refugee for Women and Children (2009b) Strategies: Li + C + Sys* | *Ethiopia* | *Post Conflict* | *Opportunistic SV Exacerbated SV* | *Livelihood strategies for refugee women Community involvement in GBV discussions Provision of ethanol stoves* | *Community members: Men and women* | *Qualitative interviews* | *Community members: Women Personnel* |
| UNHCR (1998) *Strategies: S + C + Sys + P* | Tanzania | Post- conflict | Opportunistic SV SEA Exacerbated SV | Medical/psychosocial care Community awareness raising and problem solving Health worker training Increased police presence | Whole community Survivors of SV Personnel | Implementation description/data | N/A |
| Human Rights Watch (2003) *Strategies: S+ C + Sys + P* | Nepal | Post- conflict | SEA | Improved medical protocol and response for survivors Reporting system Increased security &field staff CoC amended Livelihood program Community awareness raising | Whole community | Field visits | Women: Community members; Survivors of SV: Women; Personnel |
| Kavira & Biruru (2004) *Strategies: S + C + Li + Infrastructure (I)+ Le* | DRC | Post- conflict | Militarised SV | Medical/ psychosocial care & legal support. Support groups for SV survivors Training of health staff Community awareness raising on SV and rights -Community leader training Microfinance Provision of housing and building materials | Community members: Women Survivors of SV and children born as a result of SV | Implementation description/data | N/A |
| *O UNHCR (1997) Strategies: S + C + Sys* | *Tanzania* | *Post- conflict* | *Opportunistic SV SEA* | *Volunteers trained for first response to SV Community consultation/ awareness raising Firewood patrols & distribution* | *Whole community Survivors of SV* | *Implementation description/data* | *N/A* |
| Mabuwa (2000) *Strategies: S + C + P + Sys + I + Le* | Tanzania | Post- conflict | Opportunistic SV Exacerbated SV | Increased coordination Lawyers to support prosecution Police deployment Volunteer patrols Shelters | Survivors of SV Personnel Perpetrators/ potential perpetrators | Qualitative interviews Multiple data case studies | Not specified |
| **LEGAL STRATEGIES** | | | | | | | |
| *O Brouneus (2008)* | *Rwanda* | *Conflict* | *Militarised SV Opportunistic SV* | *Rwanda Gacaca Courts (local village tribunals adapted to address war crimes)* | *Survivors of SV* | *Qualitative interviews (n=16)* | *Survivor witnesses (involved in legal action)* |
| *O Human Rights Watch Africa (1996)* | *Rwanda* | *Conflict* | *Militarised SV* | *International Criminal Tribunal for Rwanda & state prosecution*  *Training of police & judicial officers* | *Perpetrators/ potential perpetrators* | *Implementation description/data* | *N/A* |
| *O Mischkowski & Mlinarevic (2009)* | *Yugoslavia* | *Conflict* | *Militarised SV* | *International Criminal Tribunal Yugoslavia (ICTY) War Crimes Chamber-Bosnia & Herzegovina* | *Perpetrators/ potential perpetrators* | *Qualitative interviews (n=49)* | *Judges, prosecutors, Survivor witnesses* |
| *O Nowrojee (2005)* | *Rwanda* | *Conflict* | *Militarised SV* | *International Criminal Tribunal Rwanda (ICTR)* | *Perpetrators* | *Qualitative interviews* | *Survivor witnesses* |
| *O Denov (2006)* *also reported in Livelihood Strategies | *Sierra Leone* | *Conflict* | *Militarised SV* | *Truth and Reconciliation Commission (SLTRC) Special Court for Sierra Leone (SCSL)* | *Survivors of SV/ combatants* | *Field visits & interviews* | *Survivor witnesses* |
| Amnesty International (2010) | Yugoslavia | Conflict | Militarised SV | Prosecution under state law | Survivors SV | Multiple data case studies | N/A |
| *O Women's Initiative for Gender Justice (2010)* | *Global* | *Conflict* | *Militarised SV* | *International Criminal Court* | *Perpetrators & personnel* | *Implementation description/data* | *N/A* |

1. Specific strategies were applied to these groups, as distinct from “whole community” denoting single global strategy. [↑](#footnote-ref-1)
